# The possibility of critical realist randomised controlled trials

**DOI:** 10.1186/s13063-017-1855-1

**Published:** 2017-03-21

**Authors:** Sam Porter, Tracey McConnell, Joanne Reid

**Affiliations:** 1 Department of Social Sciences and Social Work, Bournemouth University, R203, Royal London House, Christchurch Road, BH1 3LT Bournemouth, UK; 2 School of Nursing and Midwifery, Queen’s University Belfast, Belfast, BT7 1NN UK

**Keywords:** RCT, Depth realism, Critical realism, Realist evaluation, Elision, Causality

## Abstract

**Background:**

Some realists have criticised randomised controlled trials for their inability to explain the causal relations that they identify; to take into account the influence of the social context of the interventions they evaluate; and to account for individual difference. However, among realists, there is controversy over whether it is possible to improve trials by making them realist, or whether realism and the philosophical assumptions underlying trials are incompatible. This paper contributes to the debate in *Trials* on this issue. The debate thus far has concentrated on the possibility of combining trial methodology with that of realist evaluation.

**Main body:**

We concur with the contention that it is not feasible to combine randomised controlled trial design with the realist evaluation approach. However, we argue that a different variant of realism, critical realism, provides a more appropriate theoretical grounding for realist trials.

In contrast to realist evaluation, which regards social mechanisms as an amalgam of social resources and people’s reasoning, critical realism insists on their distinction. It does so on the basis of its assertion of the need to distinguish between social structures (in which resources lie) and human agency (which is at least partly guided by reasoning). From this perspective, conceiving of social mechanisms as external to participants can be seen as a valid methodological strategy for supplementing the exclusive concentration of trials on outcomes.

While accepting realist evaluation’s insistence that causality in open systems involves a configuration of multiple generative mechanisms, we adopt the critical realist interpretation of the experimental method, which sees it as creating artificial closure in order to identify the effects of specific causal mechanisms. If randomised controlled trials can be regarded as epidemiological proxies that substitute probabilistic controls over extraneous factors for closed experiments, their examination of the powers of discrete mechanisms through observation of the variation of outcomes is appropriate.

**Conclusion:**

While there are still issues to be resolved, critical realist randomised controlled trials are possible and have the potential to overcome some of the difficulties faced by traditional trial designs in accounting for the influence of social context and individual interpretation.

## Background

This paper is a further contribution to the debate in *Trials* [1, 2] about the possibility of conducting ‘genuinely realist’ [2] randomised controlled trials (RCTs). However, before engaging in the debate, we should clarify what we mean when we talk about realism, and explain why we believe that it can make a worthwhile contribution to evaluation science.

Realism, in its broadest sense, is the belief that there is an external reality that exists independently of human perception. That, of course, begs the question of what that reality consists of, and there have been many different answers to this question, giving rise to numerous schools of realism. As a result, talk of a single ‘genuine’ realism is not very helpful. Indeed, to the extent that RCTs are underpinned by Karl Popper’s post-positivist philosophy, they are already genuinely realist. Popper [3] accepted ‘realism as the only sensible hypothesis (p. 42)… [W]hat we attempt in science is to describe and (so far as possible) explain reality’ (p. 40).

For Popper, the job of science was to pose hypotheses about causal relations and then to test them empirically to see whether or not relations of regular succession could be identified between the hypothesised cause and hypothesised effect. So, Popper’s reality consisted of observable things and events. This variant of realism can be described as ‘empirical realism’ or ‘actualism’ ([4], p. 57). In contrast, the realism that is being referred to here is ‘depth realism’ ([5], p. 42). The term reflects Roy Bhaskar’s argument that there is another level of reality underlying events, consisting of the mechanisms whose powers cause those events to occur ([4], p. 56). Bhaskar uses the term ‘mechanism’ to describe causal laws to underline acceptance that their powers may be latent and their effects contingent ([4], p. 49).

Among the benefits of such an approach, he argues, is that it opens up the possibility of going beyond identification to explanation, thereby enabling a more nuanced understanding of why relationships of succession between one event and another are rarely constant. In open systems, which typically have a number of mechanisms operating simultaneously, what actually happens will depend on their combination. Thus, to take a simple example, whether or not an object floats does not only depend upon its mass, but also upon the density of the fluid it is in. In other words, its position will be dependent on the particular combination of the mechanisms of gravity and buoyancy that pertains.

But why should it matter that RCTs are founded upon a successionist view of causality? What is so debilitating about basing science on an actualist ontology? Some argue that all this philosophical conjecturing is beside the point; what matters is that RCTs (and the procedures and policies that they identify as effective) work. From such a perspective, theoretical ruminations such as this are decidedly unhelpful [6]. In a similar vein, Scriven [7] argues that the realist aspiration to explain causal relations involves a fundamental misunderstanding of the aim of evaluation, which should be purely about identifying them.

Yet, while the RCT may be lauded as the least worst way to gain accurate and unbiased knowledge about the effectiveness of interventions [8] it displays generally accepted weaknesses in its ability, for example, to generalise to different contexts, to be sensitive to individual characteristics, and to be able to discern the specific effects of components within complex interventions [9–12]. Strategies have been developed to deal with these issues, the most notable being the British Medical Research Council’s Framework for the Development and Evaluation of Complex Healthcare Interventions [13]. The MRC Framework seeks to supplement the standard RCT with procedures that take complexity and context into account. Thus, its pre-clinical and modelling phases may incorporate qualitative testing to enable investigators to describe the component parts of the intervention to ascertain which are considered essential and which can be adapted to fit with the local context. It also recommends post-trial evaluation to uncover how context influences outcomes in order to aid everyday clinical implementation.

Depth realists have pointed to the incompatibility of these adjustments with the basic philosophical assumptions on which trials are based [14, 15]. RCTs are founded on a conception of causality as the regular succession of events in the form of stimulus and response. The addition of human interpretation and choice that is implied in the adoption of qualitative methods, along with an acceptance of the role of context in explanation, undermines that conception [16]. Conversely, if the validity and reliability of RCTs is accepted, then individual perspectives and contextual influences are rendered superfluous.

From a depth realist perspective, the MRC Framework might be seen as an instance of what Kuhn [17] describes as the ad hoc adjustments made to a paradigm at the point when its inability to cope with anomalies between its theoretical foundations and the problems it faces is becoming increasingly evident [16].

Conversely, from a post-positivist perspective, developments like the Framework might be regarded as facilitating a sustainable adjustment to the methods used to evaluate complex interventions, enabling them to combine attention to the causative characteristics of both the interventions themselves and the contexts within which they occur. In other words, to return to the pragmatic assertion of the irrelevance of theory, if the Framework works, that is what matters, not its nonconformity to some obscure theoretical strictures.

The depth realist response to such pragmatic objections is that confusion in theory leads to confusion in practice. If there is ambiguity about the relationship between qualitative and quantitative data and/or between intervention and context, then it becomes difficult for researchers to construct coherent explanations [16].

However, while depth realists may be united in their dissatisfaction with RCTs’ concentration on the succession of events at a cost to examining the causes of those successions, especially when RCTs are concerned with the evaluation of interventions designed to change people’s behaviour, they are not agreed on the solution to the problem. Some regard trials as incompatible with depth realism [18]; while others argue that it is possible to conduct trials using realist assumptions [19]. The current debate in the pages of *Trials* is located within this controversy.

## Introduction

Our central contention in this paper is that realist RCTs designed to evaluate the effectiveness of behaviour-changing interventions are possible, but only if they are founded upon a different type of realist philosophy to that currently adopted by both Jamal et al. [1] in their commendation of realist RCTs and Van Belle et al. [2] in their critique of them. The common ground they share is the version of realism that is found in the philosophical underpinnings of Pawson and Tilley’s [20] realist evaluation. The problem for Jamal et al., as Van Belle et al. point out, is that the assumptions embedded in realist RCTs about the nature of causal mechanisms and the processes of causality are inimical to those of realist evaluation.

While Pawson and Tilley accept Bhaskar’s depth realism, they reject his extrapolation of its principles to the examination of the social world in the form of critical realism [15]. Critical realism, as formulated by Bhaskar [21] and developed by Archer [22] and others, regards structured social relations as possessing causal powers similar to natural causal powers, in that they are experienced by people as external forces that enable or constrain how they act. The effects of these structural mechanisms can be seen in empirically observable patterns, such as the association of social class with educational attainment. However, unlike natural mechanisms, with their wider range of influence, social mechanisms act specifically on human beings. This is significant because human beings have causal powers of their own. They have the power to choose how to act on the basis of their interpretations of what is the best course for them to take. For example, it is perfectly possible (though less likely and more difficult) for a working-class person to attain a higher degree. So, for critical realists, the human sciences have to take account of two distinct sources of causation – social structures and human agents.

In contrast, realist evaluation rejects the claim that social structures are separate, objective entities, seeing social mechanisms as the latent powers and capacities of individuals [2]. It is this interpretation of social mechanisms as having internal, reflexive components that leads realist evaluators to regard attempts to understand human behaviour through the identification of successions of events as inappropriate.

In this paper, accepting the incompatibility of RCTs with realist evaluation, we will contend that the adoption of a critical realist approach, as defined by Bhaskar [21] and Archer [22], reopens the space for realist RCTs. To support our argument, we will present the critical realist view of causal mechanisms as objective influences on human behaviour as an alternative to realist evaluation’s conception of mechanisms as combinations of resources and reasons. We will also examine how critical realist interpretations of experimental closure distinguish it from realist evaluation’s position on the focus of scientific research.

### The critique of realist RCTs

In their commentary, Van Belle et al. [2] raise at least four major objections to Jamal et al.’s [1] overview of realist RCTs, which we will address in turn. Their first is that Jamal et al.’s conception of interventions largely ignores the role of human volition in behavioural change.

Secondly, they contend that Jamal et al. regard mechanisms as external factors introduced into a social context, rather than being ‘a function of the interaction between intervention resources and responses of participants’ (p. 3).

Van Belle et al.’s third argument is that Jamal et al.’s segmentation of mechanisms into discrete, statistically amenable entities is inimical to the configurational approach to causation of what they term ‘scientific’ realism, a descriptor which we interpret as analogous to ‘depth’ realism.

Their fourth objection concerns RCTs’ requirement for randomisation and control, which means they have limited powers to deal with dynamic and complex causation.

### Neglect of volition?

Van Belle et al. [2] take issue with Jamal et al.’s [1] description of realist analysis as testing ‘how the intervention theory of change interacts with context’, arguing that it is ‘not the “intervention theory of change” that interacts with context. Rather, scientific realism holds that interventions take place in specific contexts and address actors, who decide (or not) to change their behaviour, choices or decisions in response to the resources and opportunities offered by the intervention’ (p. 2). The relationship between the three main components highlighted by Van Belle et al. might be best illustrated by an example. In a realist evaluation of the Liverpool Care Pathway for the Dying Patient [23], we noticed that the effectiveness of the pathway (intervention) was undermined by the withdrawal of educational resources to train staff in its use (context), but different actors responded to this challenge in different ways (agency), with nurses tending to continue promoting the pathway through informal education, while physicians were less committed to sustaining it.

Van Belle et al.’s charge is that Jamal et al.’s description of the engine of behavioural change confines itself to the ‘external’ causal forces contained in the intervention and its social context, ignoring the capacity of people to choose how to respond to those external forces. In other words, it is concentrating on social structures at the cost of human agency.

To the extent that Jamal et al. make no mention of human agency in their description of contexts or mechanisms, there is some merit to this charge. However, elsewhere they include qualitative process evaluation in their framework to capture ‘a sense of research participants’ own meanings, their sense of agency and how this inter-relates with the social structure of intervention context’ ([1], p. 6). While we would take issue with the confinement of Jamal et al.’s analysis to the connection between agency and context, and argue that it should be extended to include the crucial relationship between people and interventions, it is clear that their model does address human agency. However, by analytically distinguishing agency from social structure, it does so in a manner that Van Belle et al. do not perceive as legitimately realist.

### Resources and responses

Van Belle et al. argue that Jamal et al.’s description of mechanisms involves a categorical error in that it ‘wrongly implies that mechanisms can be introduced into a situation and are thus external; scientific realism holds that mechanisms are not external factors but latent powers and capabilities, which are a function of the interactions between intervention resources and responses of participants’ ([2], p. 3).

We have to take issue with Van Belle et al.’s claim that all versions of ‘scientific’ realism hold that mechanisms are a function of the interaction of resources and responses, and point to critical realism’s contrary stance, based on its separation of social structures and human agency ([21], p. 79), that the mechanisms involved in resources and responses are distinct ([22], p. 89).

#### Social structures

For critical realists, social structures possess generative mechanisms that influence the behaviour of humans, exerting ‘an objective influence which conditions action patterns and supplies agents with strategic directional guidance’ ([22], p. 196). They explain the objective influence of social mechanisms by recourse to a relational conception of society which holds that it is characterised by a matrix of relatively enduring social relations between individuals and groups (for example, between men and women or employers and employees), and that an individual’s position in this matrix will configure their opportunities or constraints, and the resources or deprivations that they experience. It will also impose cultural or legal expectations for an individual or group to engage in the practices associated with their social position(s).

#### Human agency

Social mechanisms do not act upon inanimate objects, but on human beings with the powers of reflexivity, reason and interpretation. The significance of this is that, when considering the behaviour of people, the additional component of conscious intent needs to be added to causal explanations. In many circumstances, people have the capacity to choose how they respond to external stimuli. This means, among other things, that their responses to their positions in structured social relations will not be determined by those positions, but will result from their reflection on the options that they have. More specifically, it means that their responses to changes in their position resulting from the introduction of complex interventions will be a consequence of the choices they make.

In summary, critical realism’s ‘analytical dualism’ ([22], p. 15), whereby it distinguishes social structures from human agency, entails a categorical distinction between social resources and individual reasons.

### Conflations of structure and agency

In her critique of approaches to the relationship between structure and agency that seek to identify a unitary explanation for human behaviour, Margaret Archer [22] identifies three types of conflation. ‘Downwards conflation’ is entailed in collectivist theories that place the primary causal onus on social structures and strip human choice and agency of power. ‘Upwards conflation’ can be found in social theories that insist that reality consists of nothing but individuals and their activities, giving social structures a fictional status. Critical realists reject both of these conflations on the grounds that, because both social structures and human agents possess their own unique generative mechanisms, one cannot be reduced to the other.

Traditional RCT methodology is implicitly predicated upon a downwards conflation of causal explanation in that, by regarding the intervention as the explanatory variable, it privileges structural influence over individual choice. Conversely, given RCTs’ reliance on statistical averages to identify correlations, the method is simply not designed to take individual volition into account. However, Jamal et al.’s proposal for the inclusion of qualitative empirical strategies to take account of the role of agency in the production of outcomes means that the same charge cannot be laid at the door of their conception of realist RCTs.

The third form of conflation, ‘central conflation’, differs from the first two in that it rejects the reduction of either structure or agency in favour of their mutual unification, arguing that they are inseparable and can only be conceptualised in relation to each other in a ‘duality of structure’ [24]. ‘Rather than seeing action and structure as counter-acting elements of a dualism, we should regard them as the complementary terms of a duality’ ([25], p. 58). It is not a matter of trying to explain human behaviour through the influence of external factors (as the traditional RCT does), or of explaining behaviour entirely in terms of individual volition (as much qualitative analysis does), but of appreciating ‘how action is *structured* in everyday contexts and how the structured features of action are, by the very performance of an action, thereby *reproduced*’. ([25], p. 56)

Critical realists reject the central elision of structure and agency on both ontological and methodological grounds. At an ontological level, Bhaskar [21] emphasises ‘the importance of distinguishing, in the most categorical way, between human action and the social structure’:

The properties possessed by social forms may be very different from those possessed by the individuals upon whose activity they depend. I want to distinguish sharply then between the genesis of human actions, lying in the reasons, intentions and plans of human beings, on the one hand; and the structures governing the reproduction and transformation of social activities, on the other (p. 79).

Archer [22] makes the complementary argument that the reason for distinguishing between human action and social structure is ‘not simply because ontologically they *are* indeed different entities with different properties and powers, but because methodologically it is necessary to make the distinction between them in order to *examine their interplay* and thus be able to explain why things are “so and not otherwise” in society’ (p. 64).

#### Realist evaluation’s elision

While Van Belle et al. may have overstated their case when they argued that ‘scientific realism holds that mechanisms are … a function of the interactions between intervention resources and responses of participants’, their earlier statement that ‘wide agreement exists that “response to resources” is the defining feature of mechanisms in the work of Pawson and Tilley’ ([2], p. 2) can be accepted without contention. Their critique of Jamal et al.’s separation of social resources from people’s responses is founded on the particular grounds of Pawson and Tilley’s interpretation of realism.

Pawson and Tilley [20] derive their conception of programme mechanisms as involving ‘an interplay between social resources and participants’ reasoning’ (p. 75) from their understanding of the composition of social mechanisms, which they define as being about ‘people’s choices and the capacities they derive from group membership’ (p. 66). Pawson and Tilley’s definition of social mechanisms in general, and programme mechanisms in particular, as consisting of a combination of structure (‘the capacities derived from group membership’/‘social resources’) and agency (‘people’s choices’/‘participants’ reasoning’) stands in stark contrast to the critical realist insistence that agency and structure are different entities with different properties and powers [26].

Ironically, despite their stated adoption of the assumptions underlying realist evaluation, Jamal et al.’s distinction between agency and social structure is more compatible with the assumptions of critical realism. Their interpretation of social mechanisms as objective entities that are analytically distinguishable from human agency has the benefit of enabling evaluators to examine structure and agency’s ‘interplay and thus be able to explain why things are “so and not otherwise”’ ([22], p. 64).

It has to be conceded that Jamal et al. do not fully articulate the relationships between the basic components addressed by realist RCTs. A clearer definition of those relationships is, therefore, required. To that end, we commend the model that conceives of observed outcomes as the result of the interaction of contextual mechanisms, programme mechanisms and human agency [26].

### Configuration or correlation?

Van Belle et al. argue that, in line with notions of causation traditionally associated with RCTs, Jamal et al. adopt a successionist mode of explanation which involves the correlation of variables, whereby one (the dependent variable) succeeds the other (the explanatory variable). As Van Belle et al. point out, realism rejects successionist modes of causal explanation. It does so on the grounds that they confuse causes with effects. In contrast, depth realists categorically distinguish empirical regularities from the mechanisms that generate them [4]. However, this position does not warrant Van Belle et al.’s rejection of the concept of ‘variables’, at least as it pertains to outcome patterns. Because these patterns can be varied by the introduction of an intervention, and because they are distinct from the mechanisms that generate them, Jamal et al.’s focus on their measurement does not necessarily entail the adoption of an approach ‘that reduces mechanisms … to mere variables’ (p. 4), though their use of descriptors such as ‘mediating variables’ for mechanisms seems to reflect a vestige of successionist assumptions.

While Van Belle et al. accept that Jamal et al.’s model involves multiple mechanisms, they argue that the latters’ discrete treatment of each hypothesised mechanism fails to take account of the depth realist tenet that outcomes are the result of causal configurations. This means that realism’s ‘explanation relies on showing the *relationship* between context and mechanism’, rather than treating them as separate strands (p. 4).

Van Belle et al.’s assertion that outcomes result from causal configurations is a commonly accepted tenet of depth realism, so far as it pertains to open systems. However, while causation in open systems is complex, this does not mean that individual mechanisms are bereft of distinctive causal powers. Here, we concur with Van Belle et al.’s assertion that generative mechanisms are not variables in that, while variation of the configuration of mechanisms will lead to variable outcomes, this does not imply a variation in the intrinsic qualities of the constituent mechanisms. Mechanisms’ possession of specific causal properties means that, assuming they can be isolated from other mechanisms, their powers can, in principle, be measured. From a critical realist perspective, this is the focus of experimental science, and the reason why it has been so successful [4].

As has been previously been pointed out by the defenders of realist RCTs [27] in response to the accusation that RCT design is inherently positivist [18], methods do not make assumptions, researchers (and we might add research methodologists) do. If we consider Bhaskar’s seminal depth realist examination of the experimental method in natural science [4], we can see that the purpose of experimental closure is to prevent as many mechanisms as possible, apart from the one whose causal powers are the object of interest, from exerting a differential influence, so that any regularities observed can be interpreted as being caused by the mechanism of interest. It is through this simplification of context that experimental science is able to identify particular causal powers. Thus, experimentation entails creating an artificially controlled environment with the aim testing hypotheses about specific causal mechanisms. In other words, it is concerned with neutralising configurations as far as possible, rather than treating them as the objects of investigation per se.

While Bhaskar perceives the social world as necessarily open and, therefore, not amenable to experimental closure [21], it has been argued that RCTs can be regarded as analogous to natural science experiments in that randomisation, blinding and recruitment of sufficient numbers to ensure statistical power involves the use of probability theory to approximate the closed system of the experiment [16]. To the extent that RCTs can be regarded as epidemiological proxies that substitute probabilistic controls over extraneous factors for experimental closure [28], the strategy of developing hypotheses about specific mechanisms and using statistical methods to test them would seem to be a viable course to take.

This equation of RCTs with experiments is not entirely straightforward in that, while RCTs can control for context, their focus tends to remain on the intervention, rather than specific mechanisms within the intervention. When addressing complex interventions, which frequently consist of a number of components, this lack of specific focus can compromise explanatory power [29]. However, experimental designs have been developed to identify causal mechanisms within the ‘black box’ of interventions [30], including the causal mediation analysis [31] favoured by Jamal et al., though the degree to which they are consonant with realist tenets is yet to be fully established.

Even if we allow that RCTs have the methodological capacity to impose conditions analogous to those of the closed experiment, they contain limitations that indicate the need for additional evaluative strategies. First, trials cannot take into account the causal effects of the configuration of mechanisms that come into play once experimental conditions are relaxed. Second, the outcomes they measure are not simply caused by the social mechanisms they are examining, but by the interplay between those mechanisms and the interpretive and causal powers of human agents [16].

### Simplicity and complexity

Noting that RCTs’ requirement for randomisation and control means that they can only test a few simple context-mechanism-outcome configurations, Van Belle et al. conclude that ‘at best, then, the RCT may help us in assessing the relative contribution of mechanisms to outcome patterns if the causal configuration is uniform’ (pp. 4–5). Notwithstanding Bonell et al.’s [28] response to the contrary, we concur with Van Belle et al., but interpret this circumscribed contribution to evaluation as extremely useful. It is through such simplification that the efficacy of an intervention (relating to its performance under ideal circumstances [32] can be established. However, precisely because of their closure and control, trials are far less able to demonstrate effectiveness (relating to performance under ordinary circumstances) [32].

While the demonstration of efficacy is a necessary component of evaluation, it is not a sufficient one, in that it does not take account of the effect of open systems where other mechanisms are operating concurrently [14]. Nor, with their function of describing rather than explaining ‘demi-regularities’ [33], are RCTs able to take account of human reasoning [16]. We wish to argue that these weaknesses in the experimental approach indicate the need for three distinctive methodological strategies, ‘one designed to enumerate outcome patterns; one designed to identify the mechanisms embedded in an intervention and its social context; and one designed to uncover the experiences, interpretations and responses of the actors involved’ ([34], p. 78).

RCTs can constitute one of these three strategies, in that they can be used to test efficacy by enumerating outcome patterns in tightly controlled environments which minimise (or cancel out) the effects of contextual mechanisms on outcomes [14, 16].

The second strategy involves the development and testing of realist hypotheses about the resources and restrictions embedded in the intervention and its contexts, and their relationships. This strategy can be used to build middle-range theories about processes of causation; model the intervention; refine theories to provide explanations concerning what works for whom in what circumstances; and inform those introducing efficacious interventions into open contexts of contextual mechanisms that may affect their sustained effectiveness [26].

The third strategy involves qualitative investigation designed to uncover how the human agents affected by the intervention respond to the resources and restrictions it presents. We contend that this qualitative stage should not confine itself to examination of the reasoning behind agents’ responses for the purpose of optimising the motivational appeal of the intervention. In addition to this instrumental function, it should also attempt to illuminate people’s experiences of the intervention for the purpose of critically establishing its beneficial or detrimental effects upon the lives of those affected by it [26].

## Conclusion

While our main aim has been to open up the possibility of realist RCTs as a useful approach to the evaluation of complex interventions, we do not wish push our arguments too far. There are number of reasons for caution. First, as Van Belle et al. point out, there is a dearth of practical examples of realist RCTs; even the exemplar presented by Jamal et al. is incomplete. There is a lot of practical and theoretical work yet to be done before a fully-fledged model of realist RCTs is developed. Not least, we suspect that such a model will need to be considerably lighter of touch than that currently proposed by Jamal et al. if it is not to be prohibitively burdensome. This is a significant issue because if realist trials are to have an impact on evaluation science, they are going to have to demonstrate that they are useable as well as useful.

We also do not think that the debate about the relative merits of realist RCTs versus realist evaluations nested in RCTs (or perhaps better, RCTs nested in critical realist evaluations) is settled yet. In our own empirical work relating to palliative care, we have been adopting the three parallel strategies outlined above [35–38].

Finally, given that the experimental approach to social systems that RCTs entail is not warranted by Bhaskarian realism [8], which regards those systems as irreducibly open, our mapping of RCTs onto critical realism is not straightforward. Further conversation about this extension of naturalistic science into the social world is required.

Notwithstanding these notes of caution, our purpose in this paper has been to establish the possibility and indeed desirability of realist RCTs. In terms of possibility, we hope that our discussion about the articulation of the assumptions embedded in realist RCTs with the tenets of critical realism has demonstrated that trials can be grounded in a cogent and consonant realist philosophy of science.

In terms of desirability, we have identified critical realist RCTs’ potential to overcome the limitations of traditional trial designs in accounting for the influence of social context and individual interpretation on the outcomes of complex interventions. What critical realism promises is a framework to allow researchers to combine output, process and experiential data in a complementary fashion that gives due regard to each piece of the explanatory jigsaw.

## Abbreviation

RCT: Randomised controlled trial
